# Factors associated with resection weight and complications after massive-weight-loss abdominoplasty: A retrospective 10-year single-center study

**DOI:** 10.1016/j.jpra.2026.06.010

**Published:** 2026-06-27

**Authors:** Sabrina Luettschwager, Patrick Mandal, Lars- Peter Kamolz, Hanna Luze, Maximilian Moshammer, Michael Schintler, Michael Kohlhauser

**Affiliations:** a Division of Plastic, Aesthetic and Reconstructive Surgery, Department of Surgery, Medical University of Graz, Graz, Austria; b COREMED – Center for Regenerative Medicine and Precision Medicine, JOANNEUM RESEARCH Forschungsgesellschaft mbH, Graz, Austria; c Research Unit for Tissue Regeneration, Repair and Reconstruction c/o Division of Plastic, Aesthetic and Reconstructive Surgery, Department of Surgery, Medical University of Graz, Austria; d Research Unit for Responsible Aesthetics c/o Division of Plastic, Aesthetic and Reconstructive Surgery, Department of Surgery, Medical University of Graz, Austria; e Research Unit for Safety in Health c/o Division of Plastic, Aesthetic and Reconstructive Surgery, Department of Surgery, Medical University of Graz, Austria

**Keywords:** Abdominoplasty, Resection weight, Complications, Clavien–Dindo, Post-bariatric surgery, Massive weight loss (MWL)

## Abstract

**Background:**

Abdominoplasty removes redundant skin and subcutaneous tissue but poses perioperative challenges. Although higher resection weight has been linked to complications, variation in resection burden and its relationship to post-operative complications remain incompletely defined. This exploratory study assessed patient- and procedure-related factors associated with resection weight and evaluated whether resection weight was associated with post-operative complications.

**Methods:**

This retrospective cohort included 217 adults undergoing abdominoplasty after massive weight loss (MWL; excess weight loss ≥ 50%) at the Medical University of Graz (2012–2023). Pregnancy-related and oncologic procedures, isolated revisions, and incomplete records were excluded. Variables included weight history, resection weight, post-operative complications (Clavien–Dindo), demographics, and procedure-related factors. Correlation, group comparison, multivariable linear and logistic regression analyses were applied.

**Results:**

Mean age was 45 years (SD 12); 76% were female. Mean pre-operative BMI was 26.3 kg/m^2^ (SD 3.5) and mean resection weight was 1,727 g (SD 1,132). Pre-operative BMI showed the strongest bivariate association with resection weight (Spearman r = 0.620, p < 0.001). In multivariable linear regression, pre-operative BMI (B = 209.9 g per kg/m², p < 0.001), female sex (+363.5 g, p = 0.004), metabolic disease (+346.0 g, p = 0.006), and concomitant belt lipectomy (+365.6 g, p < 0.001) were associated with higher resection weight, explaining 56% of variance. Post-operative complications occurred in 76 patients (35.0%). In adjusted logistic regression, younger age (OR 0.969 per year, p = 0.016) and pre-operative anemia (OR 2.153, p = 0.043) were associated with the composite complication endpoint; resection weight was not (OR 1.015 per 100 g, p = 0.245).

**Conclusion:**

Pre-operative BMI was the strongest factor associated with resection weight, with female sex, metabolic disease, and procedural extent adding explanatory value. Resection weight was not significantly associated with composite, major, or wound-specific complications; however, smaller effects could not be excluded.

**Abbreviations**
ASA: American Society of Anesthesiologists (classification)
BMI: Body mass index
CI: Confidence interval
EWL: Excess weight loss
FDL: Fleur-de-lis
MWL: Massive weight loss
MDE: Minimum detectable effect
OR: Odds ratio
R^2^: Coefficient of determination
SD: Standard deviation
VIF: Variance inflation factor
β: Beta coefficient
BMI pre-op: Pre-operative body mass index
BMI max: Maximum body mass index
BMI delta: Maximum body mass index – pre-operative body mass index
IQR: Interquartile range

## Introduction

Body contouring surgery, particularly abdominoplasty, is increasingly performed after massive weight loss (MWL) following bariatric surgery or lifestyle changes.^1^^,^^2^ Abdominoplasty removes redundant abdominal skin and subcutaneous tissue and is among the most common plastic surgery procedures worldwide.^3^, ^4^, ^5^ Approximately 1.2 million abdominoplasties were performed globally in 2023.^6^ This trend parallels the obesity burden: in 2022, more than 2.5 billion adults were overweight, including nearly 900 million with obesity (body mass index [BMI] ≥ 30 kg/m²).^7^

Redundant skin after MWL affects up to 96% of post-bariatric patients and imposes physical and psychosocial burden.^8^, ^9^, ^10^ Abdominoplasty addresses these issues but carries a higher complication rate than other body contouring procedures.^11^ Prior studies have linked resection weight, BMI, and method of weight loss to post-operative outcomes.^12^, ^13^, ^14^ However, reported risk estimates and proposed resection-weight threshold (commonly ≥ 2 kg) vary across studies, reflecting differences in case mix and study design.^15^, ^16^, ^17^

Although multiple predictors of complications have been described, a clinically applicable, multivariable estimate of expected resection weight, integrating pre-operative anthropometry, sex, comorbidity, and procedural extent, has not been systematically evaluated. The relative contribution of maximum BMI, pre-operative BMI, and delta BMI (maximum BMI – pre-operative BMI) remains uncertain.

The aims of this exploratory retrospective cohort study were (i) to assess patient- and procedure-related factors associated with resection weight and (ii) to evaluate its adjusted association with post-operative complications. Additional analyses explored differences by weight loss method and procedural extent, potentially supporting surgical planning, counselling and benchmarking.^18^

## Patients and methods

### Study design

This single-center retrospective study was conducted in accordance with the Declaration of Helsinki and reported using the STROBE guidelines. Ethical approval was granted by the Ethics Board of the Medical University of Graz (EK-Nr.: 36-118 ex 23/24).

### Patients and data collection

We identified 642 consecutive adults (≥18 years) who underwent abdominoplasty at the Clinical Department of Plastic, Aesthetic and Reconstructive Surgery, Graz, between 1 January 2012 and 31 December 2023. Inclusion required surgical treatment of a symptomatic abdominal panniculus after MWL, defined as excess weight loss (EWL) ≥50% (excess weight = maximum body weight – weight at BMI 25 kg/m^2^).^19^ Indications included functional impairment (e.g., recurrent intertrigo, hygiene difficulties, limitations in daily activities) or clinically relevant contour deformity after weight stabilization. Only patients with Austrian statutory insurance were included for documentation consistency.

**Fig. 1:**
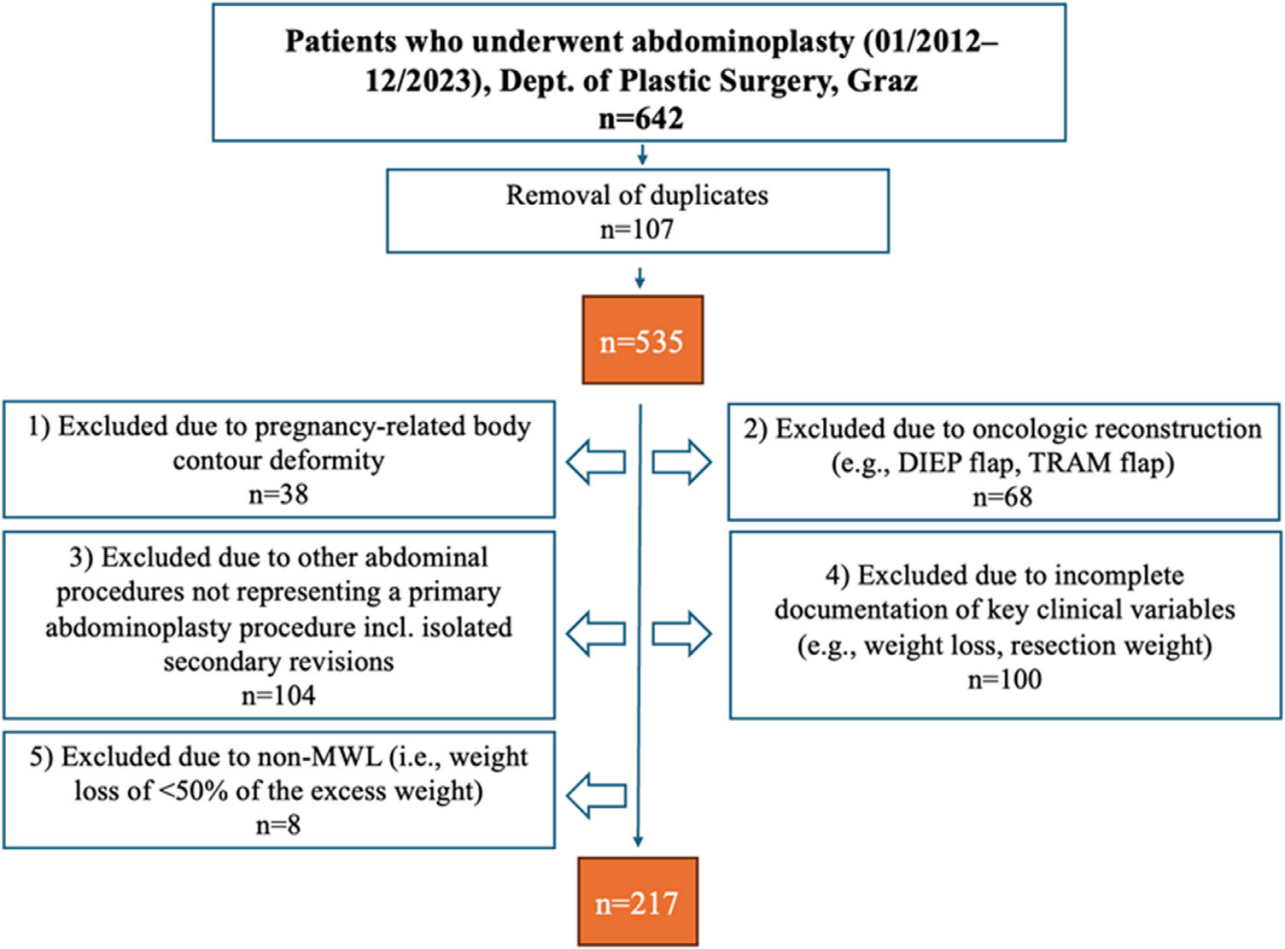
Flowchart of patient selection.

Exclusion criteria were pregnancy-related abdominoplasty, oncologic reconstruction (e.g., DIEP, TRAM), isolated secondary procedures (herniotomy, scar revision, contour refinement), incomplete documentation of key variables, and EWL <50%. Of 642 patients screened, 217 met all criteria (Fig. 1).

MWL = Massive weight loss.

### Variables

Data were retrieved from medical and surgical reports, inpatient and outpatient records, and pre-operative anesthetic documentation. Patient-related variables comprised age (years), biological sex, maximum BMI, pre-operative BMI, and delta BMI, total weight loss (kg), weight-loss duration (years), and weight-loss method (post-bariatric vs. non-bariatric).

Clinical covariates included ASA score, smoking status, metabolic disease (diabetes, hypertension, hypercholesterolemia, dyslipidemia, or hepatic steatosis), diabetes, cardiovascular disease, previous abdominal surgery, anticoagulation, steroid use, pre- and post-operative anemia (hemoglobin <12 g/dL in females and <13 g/dL in males), and operative time (minutes).

Resection weight was defined as the specimen weight attributable exclusively to the abdominoplasty component, measured intra-operatively after en-bloc excision and before wound closure. In combined procedures, only the abdominoplasty specimen was recorded. Liposuction aspirate was excluded; residual tumescent fluid may have affected measurements to an undetermined but likely small degree.

Complications were classified using Clavien–Dindo and captured during the inpatient stay and all documented follow-up at the study center (median follow-up 3 months).^20^ Complications managed elsewhere were not systematically captured.

Wound-specific complications comprised wound healing disorder or dehiscence, seroma, hematoma or post-operative bleeding, skin or fat necrosis, wound infection, fistula or suture-related complications, and medically indicated wound revision, debridement, or necrosectomy.

Complications were analyzed at the patient level using the highest applicable Clavien–Dindo grade per patient. Purely aesthetic revisions, dog-ear corrections, contour irregularities, scar revisions without wound pathology or functional impairment, and isolated hernia findings without wound-related intervention were not classified as post-operative complications.

Complication documentation was reviewed from operative reports, wound documentation, and follow-up notes. The primary complication classification was performed by a single physician investigator based on the predefined criteria. An independent physician reviewed anonymized complication descriptions while blinded to the initial classification and patient- and procedure-related variables. Exact agreement was 213/217 cases (98.2%). Cohen’s κ was 0.960 for the binary endpoint, and linear weighted κ was 0.951 (95% bootstrap CI, 0.897–0.990) for ordinal grading. Four discrepant aesthetic or contour-related revisions were resolved by consensus as non-complications. Weight loss duration was missing in 103/217 (47%) and was excluded from multivariable models; all other variables had <5% missingness and were analyzed using complete case analysis.

### Surgical technique

All procedures were performed under general anesthesia under board-certified plastic-surgeon supervision. The standard procedure was transverse abdominoplasty with umbilical transposition; a fleur-de-lis (FDL) variant was performed for pronounced vertical excess (n = 18; 8%). Rectus diastasis was corrected by midline plication when present (n = 44; 20%). Redundant skin and subcutaneous tissue were excised en bloc. Concomitant procedures included belt lipectomy (n = 115; 53%), thigh lift (n = 55; 25%), breast lift (n = 13; 6%), thoracic lift (n = 2; 0.9%), brachioplasty (n = 2; 0.9%), and lower leg lift (n = 1; 0.5%), with overlap. Procedural heterogeneity was addressed by covariate adjustment and an isolated abdominoplasty sensitivity analysis. Two closed-suction abdominal drains were used in 97% of cases.

### Statistics

Analysis was performed in IBM® SPSS® (v29; IBM Corp., Armonk, NY, USA). Continuous variables are presented as mean ± standard deviation (SD) or median with interquartile range (IQR). Because several continuous variables were non-normally distributed, Spearman correlations and Mann–Whitney U tests were used as appropriate; chi-square tests were used for categorical comparisons.

Factors associated with resection weight were assessed using multivariable linear regression. The primary model included pre-operative BMI, age, biological sex, metabolic disease, and belt lipectomy, selected based on clinical plausibility. Collinear anthropometric variables were not entered simultaneously; an alternative model substituted pre-operative weight for pre-operative BMI. Assumptions were assessed using residual and Q-Q plots, variance inflation factors (VIFs), Cook's distance, and leverage. Log-transformed resection weight was examined in sensitivity analysis. Estimates are reported with 95% confidence intervals (CI) and model fit with R^2^, adjusted R^2^, and the F-test.

Candidate variables associated with post-operative complications were screened using crosstabs and univariable logistic regression. Resection weight was retained a priori as the predefined hypothesis variable. Age and pre-operative anemia were selected based on clinical plausibility and univariable signal strength; all screened variables are reported in Table 8a. Resection weight was scaled per 100 g. The composite and wound-specific models include resection weight, age and pre-operative anemia. The major complication model included resection weight, age and pre-operative BMI; major complications were defined as Clavien–Dindo grade ≥ IIIa and occurred in 47 patients. An isolated abdominoplasty sensitivity analysis was performed.

Given the retrospective design and fixed cohort, analyses were interpreted as exploratory associations rather than confirmatory tests or definitive prediction models. Tests were two-sided, and p < 0.05 was considered statistically significant. No a priori sample size calculation was performed. Model precision was contextualized using minimum detectable effect (MDE) analyses. For the linear models, the smallest partial effect detectable with 80% power at α = 0.05 was f² = 0.037. For the logistic models, MDE odds ratios for resection weight per 100 g were estimated using the method of Hsieh, Bloch and Larsen.^21^ Calculations were based on the observed standard deviation of resection weight and an auxiliary R^2^ of 0.20.

## Results

### Cohort characteristics

Of 642 patients screened, 217 met inclusion criteria (Fig. 1). Mean age was 45 ± 12 years; 164 (76%) were female. Mean pre-operative BMI was 26.3 ± 3.5 kg/m², maximum BMI 43.1 ± 6.8 kg/m², and delta BMI 16.8 ± 5.9 kg/m². Eighty-five patients (39%) had undergone bariatric surgery, and 132 patients (61%) achieved non-surgical weight loss. Mean weight loss was 48 ± 17 kg, and mean resection weight was 1,727 ± 1,132 g (range 87–6,980 g) (Table 1). FDL was performed in 18 (8%), rectus plication in 44 (20%), quilting/progressive-tension sutures in 14 (6%), and liposuction in 22 (10%). Belt lipectomy was performed in 115 (53%); 158 patients (73%) underwent at least one concomitant procedure (Table 2a). Among the listed concomitant body-contouring procedures excluding liposuction, 66 patients (30.4%) had none, 114 (52.5%) had one, and 37 (17.1%) had two; no patient had three or more (Table 2b).

**Table 1:** Patient cohort characteristics.

	Total (N = 217)
**Age** (years)*	45 ± 12 (21–79)
**Sex**	
Female	164 (76%)
Male	53 (24%)
**BMI max** (kg/m²)*	43.1 ± 6.8 (25.8–70.2)
**BMI delta** (kg/m²)*	16.8 ± 5.9 (5.3–40.9)
**BMI pre-op** (kg/m²)*	26.3 ± 3.5 (17.3–42.6)
**Weight loss** (kg)*	48 ± 17 (15–110)
**Weight loss method**	
Non-bariatric	132 (61%)
Post-bariatric	85 (39%)
**Smoking**	
No	101 (47%)
Yes	116 (53%)
**Metabolic disease**	
No	139 (64%)
Yes	78 (36%)
**Pre-operative anemia**	
No	182 (84%)
Yes	35 (16%)
**Post-operative complication**	
No	141 (65%)
Yes	76 (35%)
Resection weight (g)*	1727 ± 1132 (87–6980)
Female	1817 ± 1200 (87–6980)
Male	1452 ± 841 (262–5000)
	Total (N = 114)
**Time period of weight loss** (years)⁎⁎	3.0 (IQR 2.0–5.0)

⁎ Values are presented as mean ± SD, with range in parentheses

⁎⁎ Values are presented as median with interquartile range (IQR) in parentheses; BMI max = maximum body mass index; BMI delta = maximum body mass index–pre-operative body mass index; BMI pre-op = pre-operative body mass index.

**Table 2a:** Abdominoplasty procedure characteristics.

	Total (N = 217)
**Fleur-de-lis**	
Yes	18 (8%)
**Rectus plication**	
Yes	44 (20%)
**Quilting/progressive tension sutures**	
Yes	14 (6%)
**Liposuction**	
Yes	22 (10%)
**Belt lipectomy**	
Yes	115 (53%)
**Thoracic lift**	
Yes	2 (0.9%)
**Brachioplasty**	
Yes	2 (0.9%)
**Breast lift**	
Yes	13 (6%)
**Lower leg lift**	
Yes	1 (0.5%)
**Thigh lift**	
Yes	55 (25%)
**Concomitant procedures**	
Yes	158 (73%)

**Table 2b:** Combinations of concomitant body-contouring procedures excluding liposuction.

**Concomitant procedure combination with abdominoplasty**	**n (% of total cohort)**
**No listed concomitant body-contouring procedure excluding liposuction**	66 (30.4%)
**Belt lipectomy only**	78 (35.9%)
**Belt lipectomy + thigh lift**	30 (13.8%)
**Thigh lift only**	25 (11.5%)
**Breast lift only**	8 (3.7%)
**Belt lipectomy + breast lift**	5 (2.3%)
**Thoracic lift only**	2 (0.9%)
**Brachioplasty only**	1 (0.5%)
**Belt lipectomy + brachioplasty**	1 (0.5%)
**Belt lipectomy + lower leg lift**	1 (0.5%)
**Total**	217 (100%)

All patients underwent abdominoplasty. Liposuction was reported separately as an adjunctive contouring procedure in Table 2a and was not included in this combination table. Therefore, “no listed concomitant body-contouring procedure excluding liposuction” does not necessarily equal isolated abdominoplasty; the predefined isolated-abdominoplasty subgroup comprised 59 patients.

### Comparison between non-bariatric and post-bariatric patients

Post-bariatric patients were older (48 ± 12 years [27–79] vs. 43 ± 12 years [21–75], p = 0.002), had longer weight loss duration (4.0 years [IQR 3.0–5.0] vs. 2.0 years [IQR 2.0–4.0], p < 0.001), higher maximum BMI (46.3 ± 6.5 kg/m^2^ [34.9–70.2] vs. 41.1 ± 6.2 kg/m² [25.8–66.9], p < 0.001), and greater delta BMI (19.6 ± 5.5 kg/m^2^ [8.9–36.8] vs. 15.0 ± 5.5 kg/m^2^ [5.3–40.9], p < 0.001). Pre-operative BMI was comparable (26.7 ± 4.1 kg/m^2^ [17.3–42.6] vs. 26.1 ± 3.1 kg/m^2^ [19.8–38.8], p = 0.456). Resection weight did not differ significantly between post-bariatric and non-bariatric patients (1,942 ± 1,288 g vs. 1,590 ± 1,000 g, p = 0.057) (Table 3).

**Table 3:** Comparison of characteristics between non-bariatric and post-bariatric patients.

	**Non-bariatric group**	**Post-bariatric group**	**p-value**
	Total (N = 132)	Total (N = 85)	0.006
**Female**	91 (69%)	73 (86%)	
**Male**	41 (31%)	12 (14%)	
**Age** (years)*	43 ± 12 (21–75)	48 ± 12 (27–79)	0.002
**Weight loss** (kg)*	44 ± 17 (15–110)	55 ± 16 (25–104)	<0.001
**BMI max** (kg/m^2^)*	41.1 ± 6.2 (25.8–66.9)	46.3 ± 6.5 (34.9–70.2)	<0.001
**BMI delta** (kg/m^2^)*	15.0 ± 5.5 (5.3–40.9)	19.6 ± 5.5 (8.9–36.8)	<0.001
**BMI pre-op** (kg/m^2^)*	26.1 ± 3.1 (19.8–38.8)	26.7 ± 4.1 (17.3–42.6)	0.456
**Resection weight** (g)*	1590 ± 1000 (87–6980)	1942 ± 1288 (323–6453)	0.057
**Time period of weight loss** (years)⁎⁎	2.0 (IQR 2.0–4.0)	4.0 (IQR 3.0–5.0)	<0.001
**Smoking**			0.577
No	59 (45%)	42 (49%)	
Yes	73 (55%)	43 (51%)	
**Metabolic disease**			0.001
No	96 (73%)	43 (51%)	
Yes	36 (27%)	42 (49%)	
**Complication**			0.419
No	83 (63%)	58 (68%)	
Yes	49 (37%)	27 (32%)	
**Anemia pre-op**			0.706
No	112 (85%)	70 (82%)	
Yes	20 (15%)	15 (18%)	

⁎ Values are presented as mean ± SD, with range in parentheses

⁎⁎ Values are presented as median with interquartile range (IQR) in parentheses; BMI max = maximum body mass index; BMI delta = maximum body mass index–pre-operative body mass index; BMI pre-op = pre-operative body mass index.

### Missing data

Missingness was low across key variables. Weight-loss duration was available in 114/217 patients (53%), whereas operative time had <2% missing data (Table 4).

**Table 4:** Missingness across key study variables.

**Total (N =217)**	**Valid n (%)**	**Missing n (%)**
**Weight loss duration** (years)	114 (53%)	103 (47%)
**Operative time** (min)	214 (99%)	3 (1%)

### Factors associated with resection weight

Bivariate correlations with resection weight were strongest for pre-operative BMI (Spearman r = 0.620, p < 0.001), followed by pre-operative weight (r = 0.458, p < 0.001), maximum BMI (r = 0.320, p < 0.001), and age (r = 0.288, p < 0.001). Delta BMI (r = -0.03, p = 0.70) and duration of weight loss (r = 0.15, p = 0.12) were not associated (Table 5).

**Table 5:** Bivariate correlations between resection weight and selected anthropometric and demographic variables.

**Variable**	**Correlation Coefficient r (Spearman)**	**p (2-tailed)**	**N**
**Age** (years)	0.288⁎⁎	<0.001	217
**Sex**	0.137*	0.044	217
**Weight max** (kg)	0.238⁎⁎	<0.001	217
**Weight pre-op** (kg)	0.458⁎⁎	<0.001	217
**BMI max** (kg/m^2^)	0.320⁎⁎	<0.001	217
**BMI delta** (kg/m^2^)	-0.026	0.704	217
**BMI pre-op** (kg/m^2^)	0.620⁎⁎	<0.001	217
**Weight loss duration** (years)	0.148	0.116	114

⁎⁎ Correlation is significant at the 0.01 level (2-tailed);

⁎ Correlation is significant at the 0.05 level (2-tailed); BMI max = maximum body mass index; BMI delta = maximum body mass index–pre-operative Body mass index; BMI pre-op = pre-operative body mass index. Because BMI delta is mathematically derived from BMI max and BMI pre-op, these variables were not entered simultaneously into the same multivariable regression model.

In the primary multivariable linear regression, pre-operative BMI showed the strongest adjusted association with resection weight (+209.9 g per kg/m^2^, 95% CI 179.9–239.9, p < 0.001). Female sex (+363.5 g, 95% CI 115.1–612.0, p = 0.004), metabolic disease (+346.0 g, 95% CI 101.0–591.1, p = 0.006), and concomitant belt lipectomy (+365.6 g, 95% CI 158.0–573.3, p < 0.001) also showed positive adjusted associations with resection weight. Age was not associated with resection weight after adjustment (p = 0.636). The model explained 56% of the variance (R^2^ = 0.557, adjusted R^2^ = 0.547, F (5,211) = 53.1, p < 0.001) (Table 6a). VIFs were ≤ 1.5. Diagnostics showed approximately normal residuals with minor upper-tail deviation, no major heteroscedasticity, and no influential observations (Cook's D ≤ 0.18). Log-transformation did not materially alter the results. Findings were comparable in the alternative weight-based model (Table 6b). The minimum detectable partial R² was approximately 3.6% for both linear models at 80% power.

**Table 6a:** Primary multivariable linear regression model of factors associated with resection weight using BMI_pre-op_.

**Predictor**	**B (g)**	**SE**	**β**	**95% CI**	**p**
Constant	−4502.9	420.0	—	−5330.8 to −3674.9	<0.001
Age (years)	2.5	5.3	0.027	−7.9 to 12.9	0.636
Female sex	363.5	126.0	0.138	115.1 to 612.0	0.004
Metabolic disease	346.0	124.3	0.147	101.0 to 591.1	0.006
Belt lipectomy	365.6	105.4	0.162	158.0 to 573.3	<0.001
BMI pre-op (kg/m²)	209.9	15.2	0.656	179.9 to 239.9	<0.001

**Model fit: R² =** 0.557, **Adjusted R² =** 0.547, **F(5,211) =** 53.10**, p < 0.001, N =** 217

* No relevant multicollinearity was observed (all VIFs ≤ 1.50); BMI pre-op = pre-operative body mass index.

**Table 6b:** Alternative multivariable linear regression model of factors associated with resection weight using pre-operative weight.

**Predictor**	**B (g)**	**SE**	**β**	**95% CI**	**p**
Constant	−5055.6	431.9	—	−5907.1 to −4204.2	<0.001
Age (years)	10.9	5.0	0.116	1.0 to 20.8	0.031
Female sex	1112.2	137.0	0.423	842.1 to 1382.2	<0.001
Metabolic disease	363.1	120.8	0.154	125.1 to 601.2	0.003
Belt lipectomy	360.7	102.4	0.159	158.8 to 562.6	<0.001
Pre-operative weight (kg)	68.1	4.7	0.736	58.9 to 77.3	<0.001

**Model fit: R² =** 0.582, **Adjusted R² =** 0.572, **F(5,211) =** 58.66**, p < 0.001, N =** 217

### Complication frequency and severity

Post-operative complications occurred in 76 of 217 patients (35.0%): 37.1% in the non-bariatric and 31.8% in the post-bariatric group (p = 0.419). Minor complications (Clavien–Dindo grades I–II) occurred in 29 patients (13.4%) and major complications (grade ≥ IIIa) in 47 patients (21.7%); no grade IV or V events occurred. The distribution of no, minor, and major complications did not differ between non-bariatric and post-bariatric patients (χ²(2) = 0.67, p = 0.714) (Table 7a). Wound-specific complications occurred in 71 (32.7%) (Table 7b).

**Table 7a:** Post-operative complications according to Clavien–Dindo classification by weight loss procedure.

	Non-bariatric group (N = 132)	Post-bariatric group (N = 85)
No complications*	83 (63%)	58 (68%)
Complications	49 (37%)	27 (32%)
Minor complications*	19 (14%)	10 (12%)
Degree I	16 (12%)	9 (11%)
Degree II	3 (2%)	1 (1%)
Major complications*	30 (23%)	17 (20%)
Degree IIIa	14 (11%)	7 (8%)
Degree IIIb	16 (12%)	10 (12%)
Degree IV	0 (0%)	0 (0%)
Degree V	0 (0%)	0 (0%)

⁎ p = 0.714, Chi-square test comparing the overall distribution of no, minor (Clavien–Dindo I–II), and major (≥ IIIa) complications between non-bariatric and post-bariatric patients.

**Table 7b:** Wound-specific complication types.

**Wound-specific complication category**	**n**	**% of total cohort**
Any wound-specific complication	71	32.7%
Wound healing disorder / dehiscence	35	16.1%
Seroma	18	8.3%
Hematoma / postoperative bleeding	15	6.9%
Skin or fat necrosis	5	2.3%
Wound infection	4	1.8%
Fistula / suture-related complications	3	1.4%
Medically indicated wound revision / debridement / necrosectomy	7	3.2%

Categories were not mutually exclusive; one patient could have more than one wound-specific complication type. Repeated occurrences of the same subtype within one patient, such as multiple seroma punctures, were counted once. Percentages refer to the total cohort of 217 patients.

### Predictors of complications

Screening identified age and pre-operative anemia as the strongest candidate associations; resection weight was retained as the predefined hypothesis variable despite no univariable association (Table 8a). In the adjusted composite endpoint model, younger age and pre-operative anemia were associated with post-operative complications (age: OR 0.969, 95% CI 0.944–0.994, p = 0.016; pre-operative anemia: OR 2.153, 95% CI 1.024–4.528, p = 0.043). Resection weight was not significantly associated with the composite endpoint (OR 1.015 per 100 g, 95% CI 0.990–1.042, p = 0.245) (Table 8b).

**Table 8a:** Screening of candidate factors for post-operative complications.

**Predictor**	**Screening method**	**Effect estimate / raw rate**	**p-value**	**Retained for model**
**Age (per year)**	Univariable logistic regression	OR 0.971	0.020	Yes
**Pre-operative anemia**	Crosstab / χ²	51.4% vs. 31.9%	0.026	Yes
**Resection weight (per 100 g)**	Univariable logistic regression	OR 1.007	0.594	Yes*
Cardiovascular disease	Crosstab + univariable logistic regression	OR 0.515; 24.6% vs. 38.8%	0.054 / 0.056	No
Post-op anticoagulation	Crosstab / χ²	23.1% vs. 37.6%	0.084	No
Sex	Crosstab / χ²	33.5% vs. 39.6%	0.419	No
Weight loss method	Crosstab + univariable logistic regression	OR 0.789; 31.8% vs. 37.1%	0.419 / 0.420	No
Smoking	Crosstab / χ²	32.8% vs. 37.6%	0.454	No
Diabetes mellitus	Crosstab / χ²	38.1% vs. 34.7%	0.756	No
Liposuction	Crosstab / χ²	50.0% vs. 33.3%	0.120	No
Belt lipectomy	Crosstab / χ²	34.5% vs. 35.6%	0.858	No
BMI pre-op (per kg/m²)	Univariable logistic regression	OR 0.996	0.912	No
Metabolic disease	Crosstab / χ²	30.8% vs. 37.4%	0.325	No
ASA Score	Crosstab / χ²	No clear gradient	0.690	No
Previous abdominal surgery	Crosstab / χ²	34.5% vs. 35.6%	0.870	No
Rectus plication	Crosstab / χ²	25.6% vs. 37.4%	0.147	No
Fleur-de-lis (FDL)	Crosstab / χ²	38.9% vs. 34.7%	0.720	No
Quilting sutures	Crosstab / Fisher	14.3% vs. 36.5%	0.146	No
Steroid use	Crosstab / Fisher	40.0% vs. 34.9%	1.000	No
Alcohol use	Crosstab / χ²	38.5% vs. 29.9%	0.194	No
Thigh lift	Crosstab / χ²	29.6% vs. 36.8%	0.338	No
Breast lift	Crosstab / Fisher	30.8% vs. 35.3%	1.000	No

⁎ Retained as the predefined primary hypothesis variable despite the absence of a univariable signal. BMI pre-op = pre-operative body mass index.

**Table 8b:** Multivariable logistic regression model for post-operative complications.

**Predictor**	**OR**	**95% CI**	**p**
Age	0.969	0.944 to 0.994	0.016
Pre-operative anemia*	2.153	1.024 to 4.528	0.043
Resection weight (per 100 g)	1.015	0.990 to 1.042	0.245

Based on 217 complete cases; pre-operative anemia was present in 35 patients (16.1%).

⁎ Reference category: no pre-operative anemia

Resection weight was not associated with major complications (OR 1.014 per 100 g, 95% CI 0.976–1.054, p = 0.471) or wound-specific complications (OR 1.018 per 100 g, 95% CI 0.992–1.045, p = 0.179) (Table 8c, Table 8d). At 80% power, the MDE odds ratios were 1.040, 1.047, and 1.041 per 100 g for the composite, major, and wound-specific endpoints, respectively. The observed odds ratios were below these thresholds in all three models.

**Table 8c:** Multivariable logistic regression model for major post-operative complications.

**Predictor**	**OR**	**95% CI**	**p**
Age	0.977	0.949 to 1.006	0.115
BMI pre-op (kg/m^2^)	1.032	0.908 to 1.173	0.626
Resection weight (per 100 g)	1.014	0.976 to 1.054	0.471

Major complications were defined as Clavien–Dindo grade ≥ IIIa. Based on 217 complete cases and 47 major events.

BMI pre-op = pre-operative body mass index.

**Table 8d:** Multivariable logistic regression model for wound-specific complications.

**Predictor**	**OR**	**95% CI**	**p**
Age	0.972	0.947 to 0.998	0.035
Pre-operative anemia*	2.139	1.015 to 4.506	0.046
Resection weight (per 100 g)	1.018	0.992 to 1.045	0.179

Based on 217 complete cases; pre-operative anemia was present in 35 patients (16.1%).

Wound-specific complications included wound healing disorder/dehiscence, seroma, hematoma/postoperative bleeding, skin or fat necrosis, wound infection, fistula/suture-related complications, and medically indicated wound revision/debridement.

⁎ Reference category: no pre-operative anemia.

### Sensitivity analyses

Restricting analyses to isolated abdominoplasty (n = 59) confirmed the pre-operative BMI (β = 177.7 g per 1 kg/m², standardized β = 0.72, p < 0.001) and metabolic disease (β = 549.0 g, standardized β = 0.26, p = 0.011) associations, whereas the female sex effect was attenuated (β = 30.9 g, standardized β = 0.01, p = 0.887). No association between resection weight and complications emerged in this subgroup (OR = 0.975 per 100 g, Nagelkerke R^2^ = 0.018, p = 0.409) (Table 9).

**Table 9:** Sensitivity analyses comparing the primary study findings with the subgroup restricted to isolated abdominoplasty procedures (n=59).

**Outcome**	**Main analysis**	**Isolated ABP**	**Interpretation**
BMI pre-op → resection weight	β = 209.9 g per 1 kg/m²; p < 0.001	β = 177.7 g per 1 kg/m²; standardized β = 0.72; p < 0.001	robust
Female sex → resection weight	β = +363.5 g; p = 0.004	β = +30.9 g; standardized β = 0.01; p = 0.887	attenuated
Metabolic disease → resection weight	β = +346.0 g; p = 0.006	β = +549.0 g; standardized β = 0.26; p = 0.011	robust
Resection weight → complication	OR = 1.015 per 100 g; p = 0.245	OR = 0.975 per 100 g; p = 0.409; Nagelkerke R^2^ = 0.018	No evidence of association

BMI pre-op = pre-operative body mass index.

## Discussion

In this exploratory cohort, pre-operative BMI, female sex, metabolic disease, and concomitant belt lipectomy were associated with resection weight after adjustment (R^2^ = 0.56), whereas resection weight was not significantly associated with composite, major, or wound-specific complications. Given the retrospective design and limited number of severity-stratified events, these findings should be interpreted as exploratory associations rather than confirmatory prediction results.

The strong association of pre-operative BMI indicates that the patient’s pre-operative anthropometric profile was more closely related to resection weight than historical weight trajectory.^22^^,^^23^ BMI delta and weight-loss duration were not associated with resection weight. However, BMI delta does not capture skin quality, tissue laxity, elasticity, or adipose distribution. No mechanistic conclusion can be drawn regarding residual adiposity versus skin properties.

Female sex was associated with higher resection weight in the primary model, consistent with reported differences in fat distribution, skin structure, and soft-tissue biomechanics.^24^^,^^25^ This association attenuated in the isolated-abdominoplasty subgroup, suggesting that possible procedural selection may partly explain the finding. Metabolic disease may reflect obesity-related tissue burden, while the association with belt lipectomy indicates that resection weight also depends on surgical strategy.

No significant association was observed across all three complication endpoints. This contrasts with prior studies linking higher resection weight or operative burden, particularly resection weights ≥ 2 kg, to increased complication rates.^16^^,^^17^ Resection weight may be an imperfect proxy for operative burden in a cohort with frequent concomitant procedures. In addition, institutional BMI thresholds and pre-operative optimization within the Austrian statutory insurance framework may have reduced the representation of patients at particularly high operative risk. Accordingly, smaller clinically relevant effects cannot be excluded.

Contrary to some prior reports,^15^ overall and severity-stratified complication rates did not differ significantly between post-bariatric and non-bariatric patients. This may reflect selection within the Austrian statutory insurance framework, including requirements for weight stability and pre-operative optimization.

The primary model yielded the following multivariable equation:

$$\begin{array}{ccc} & & \mathrm{Predicted}\;\mathrm{resection}\;\mathrm{weight}\;\left(g\right)\;=\;-4502.9\;+\;209.9\;\times \;\mathrm{pre}-\mathrm{operative}\;\mathrm{BMI}\;+\;363.5\;\\ & & \times \;\mathrm{female}\;\mathrm{sex}\;+\;346.0\;\times \;\mathrm{metabolic}\;\mathrm{disease}\;+\;365.6\;\times \;\mathrm{belt}\;\mathrm{lipectomy} \end{array}$$

Female sex, metabolic disease, and belt lipectomy are binary variables (1 = yes, 0 = no). For example, a female patient with a pre-operative BMI of 30 kg/m², metabolic disease, and planned belt lipectomy would have an estimated resection weight of approximately 2.9 kg. This point estimate should not be interpreted as an individual prediction interval. The equation requires external validation and recalibration. Given the substantial residual variance and absence of external validation, it should be considered exploratory rather than a validated clinical prediction tool.

### Clinical implications

After external validation, the equation may support pre-operative counselling, surgical planning, and institutional benchmarking by providing an estimate of expected resection weight. Such estimates could assist with case preparation and communication of the anticipated extent of resection. Future prospective studies should evaluate additional factors, including nutritional markers,^26^ body composition, smoking exposure,^27^ duration of weight stability, physical activity, and patient-reported symptom burden.

### Strengths and limitations

Strengths include the relatively large consecutive single-center cohort comprising both post-bariatric and non-bariatric patients. Additional strengths include clinically motivated multivariable modelling with diagnostics and sensitivity analyses, and standardized Clavien–Dindo grading with independent blinded review.

Limitations include the retrospective design and associated documentation bias, particularly for minor complications managed outside the center. Despite high inter-rater agreement, retrospective Clavien–Dindo grading remains subject to documentation-dependent misclassification. Median follow-up of 3 months limited the detection of late complications. The complication analyses were exploratory, and MDE analyses indicated that odds ratios below approximately 1.04–1.05 per 100 g could not be excluded, particularly for severity-specific endpoints. The wound-specific endpoint was more clinically homogeneous than the overall composite endpoint but remained mechanistically heterogeneous. The study was not powered for subtype-specific analyses.

Nutritional markers were inconsistently recorded. The reported diabetes prevalence (n = 17, 8%) is likely an underestimate attributable to underdocumentation.^28^ Weight-loss duration showed substantial missingness and was excluded from multivariable models.

Combined procedures were frequent (n = 158, 73%), limiting transferability to isolated aesthetic abdominoplasty. Belt lipectomy was included in the primary model, and isolated-abdominoplasty sensitivity analyses showed broadly consistent patterns. This small subgroup (n = 59) limited the precision of complication estimates. Less frequent concomitant procedures were not individually modelled. Residual tumescent fluid may have slightly inflated measured resection weight. Restriction to patients covered by Austrian statutory insurance further limits generalizability to reconstructive post-MWL populations.

## Conclusion

In this exploratory cohort of 217 post-MWL abdominoplasty patients, pre-operative BMI was most strongly associated with resection weight, with female sex, metabolic disease, and concomitant belt lipectomy providing additional explanatory value. BMI delta and weight-loss duration were not associated with resection weight, suggesting that current anthropometric status may be more informative than weight-loss history. Resection weight was not significantly associated with composite, major, or wound-specific complications; however, MDE analyses indicate that smaller effects cannot be excluded. The proposed equation may serve as a basis for further evaluation of resection-weight estimation. External validation is required before it can be considered for clinical use in surgical planning or patient counselling. Prospective multicenter studies are needed to confirm the resection-weight model and complication-risk findings.

## Declaration of generative AI and AI-assisted technologies in the manuscript preparation process

During the preparation of this work, the authors used ChatGPT and Claude solely to improve the language and readability of the manuscript. After using these tools, the authors reviewed and edited the content as needed and take full responsibility for the content of the publication.

## Funding

This research did not receive any specific grant from funding agencies in the public, commercial, or not-for-profit sectors.

## Ethical approval

Ethical approval was granted by the Ethics Board of the Medical University of Graz (EK-Nr.: 36-118 ex 23/24). The study was conducted in accordance with the Declaration of Helsinki.

## Declaration of competing interest

None.
